# *OsIPK2* Regulates Seed Vigor by Integrating IP_6_ Biosynthesis, Auxin Signaling, and H3K27me3 Deposition in Japonica Rice

**DOI:** 10.3390/biology15020155

**Published:** 2026-01-15

**Authors:** Yao Chen, Ya Li, Sihong Sang

**Affiliations:** 1 College of Life Sciences, Luoyang Normal University, Luoyang 471934, China; liya43320892@163.com; 2 School of Pharmacy, Guizhou University of Traditional Chinese Medicine, Guiyang 550025, China

**Keywords:** seed vigor, inositol hexakisphosphate, *OsIPK2*, auxin, epigenetic regulation, rice

## Abstract

Seed vigor is a key agronomic trait that integrates germination capacity and seedling establishment, critically influencing rice productivity. Inositol hexakisphosphate (IP_6_) serves as a major phosphorus reservoir in seeds, yet its regulatory mechanism in seed vigor remains unclear. Here, we demonstrate that exogenous IP_6_ application inhibited seed germination and seedling growth of japonica rice *(Oryza sativa* L. ssp. *japonica cv. Zhonghua11*) in a dose-dependent manner; 10 mM IP_6_ reduced seed germination by 100%, while 100 μM IP_6_ suppressed primary root length by 33.6% compared to the control. This inhibitory effect is likely mediated by antagonizing auxin signaling, as supported by suppressed *DR5::GUS* expression and altered transcription of auxin-responsive genes. *OsIPK2*, a key enzyme in IP_6_ biosynthesis, showed high expression during early development in rice. RNA interference of *OsIPK2* led to a 40.8–61.7% reduction in seed IP_6_ content, 45.3–65% higher zinc (Zn) and iron (Fe) accumulation, and a 35.4–53.5% lower germination rate compared to wild-type (WT). Conversely, *OsIPK2*-RNAi seedlings exhibited enhanced growth and resistance to IP_6_, which was associated with misregulation of auxin-responsive genes and a decrease in the repressive histone mark H3K27me3 at their loci. Furthermore, endogenous indole-3-acetic acid (IAA) levels significantly reduced in Ri-1 but unchanged in Ri-2, while abscisic acid (ABA) content and the IAA/ABA ratio remained unaltered compared to wild-type. Our findings reveal that *OsIPK2* balances seed vigor and seedling development by modulating inositol phosphate metabolism, auxin responses, and epigenetic regulation, providing insights for improving seed quality in cereals. Whether the regulatory role of *OsIPK2* in seed vigor is conserved across other rice subspecies requires further investigation.

## Simple Summary

Seed vigor is a crucial trait for crops to germinate and grow into healthy seedlings, which directly impacts crop yield. As a major phosphorus reservoir in seeds, inositol hexakisphosphate (IP_6_) is known to affect seed vigor, but the underlying mechanisms remain unclear. In this study, we found that exogenous application of IP_6_ inhibited rice seed germination and seedling growth by disrupting auxin signaling. Intriguingly, RNA interference (RNAi) of *OsIPK2*, a key enzyme in the IP_6_ biosynthesis pathway, led to reduced seed IP_6_ content and altered seed morphology, which consequently inhibited germination. Yet, the resulting seedlings exhibited enhanced growth performance. We also found that *OsIPK2* is associated with altered plant response to auxin, which correlates with changes in the histone mark H3K27me3 at auxin-responsive gene loci, potentially contributing to the fine-tuning of their transcriptional activity. These findings provide novel molecular insights to inform future genetic approaches for improving seed quality in rice.

## 1. Introduction

Seed vigor is a complex agronomic trait encompassing seed aging tolerance, dormancy, viability, rapid germination, and seedling establishment [1]. It reflects the inherent physiological activity of seeds during germination and early seedling growth [2]. Consequently, seed vigor determines the field performance and ultimate productivity of cereal crops, particularly in rice (*Oryza sativa* L.), a staple food for over half of the world’s population [3].

The seed vigor of plants is governed by the activation and utilization of endogenous stored reserves, such as carbohydrates, proteins, and lipids, which provide energy and substrates for the germination process [4]. Among these storage compounds, inositol hexakisphosphate (IP_6_, phytic acid) is the main phosphorus storage form in seeds, accounting for 1–5% of seed dry mass and 65–85% of total phosphorus [5]. During germination, phytase degrades phytate to release free phosphorus and minerals, which support early seedling growth [6]. IP_6_ is also regarded as an anti-nutritional component due to its potential cation-chelating capacity, with a high affinity for binding minerals such as iron (Fe) and zinc (Zn) [7]. Additionally, it suppresses the iron-mediated generation of reactive oxygen species (ROS) and of lipid peroxidation, thus maintaining the viability of seeds [8,9]. Beyond preserving seed viability, seed-borne IP_6_ acts as a key determinant of vigorous seedling establishment under stressful environments [10]. Low phytic acid (*lpa*) plants often exhibit impaired germination, reduced seed mass, diminished yield, and in some cases, seed lethality [11,12]. Notably, while external application of inorganic phosphorus (Pi) exerts an additive effect on enhancing seed vigor, exogenous IP_6_ treatment inhibits early seedling growth in a dose-dependent manner, as demonstrated in wheat [13]. This contradiction implies that the regulatory mechanism of IP_6_ in seed vigor is far more complex than previous understanding.

The biosynthesis of IP_6_ involves sequential phosphorylation of myo-inositol at all six hydroxyl positions by specific inositol kinases. Several mutants of IP_6_ biosynthesis genes in the inositol phosphate pathway have been reported to exhibit defects in seed vigor, in addition to low IP_6_ content. For example, the knockout mutation of inositol 1,3,4,5,6-pentakisphosphate 2-kinase gene (*IPK1*) in rice or Arabidopsis led to a lower germination rate, retarded growth, or lethality at the seedling stage [14,15]. Similarly, mutation of the inositol 1,3,4-trisphosphate 5/6-kinase 6 gene (*ITPK1*) in rice led to slower germination and poor fertility [16]. These findings collectively suggest that the integrity of the IP_6_ biosynthesis pathway, particularly the key catalytic steps mediated by enzymes, is crucial for establishing and maintaining seed vigor, ensuring successful germination and normal early seedling development in plants. However, the regulatory mechanisms linking IP_6_ metabolism to seed vigor remain poorly understood.

Intriguingly, emerging evidence suggests crosstalk between IP_6_ and phytohormone signaling pathways, which may collectively modulate seed vigor. For instance, IP_6_ or its derivatives have been reported to be ligands of multiple plant hormone receptors, such as TIR1-ASK1-AUX/IAA, and COI1–JAZ [17,18]. Yet, the specific regulatory mechanisms underlying IP_6_-mediated modulation of auxin responses remain unclear, warranting further investigation into the coordinated effects of IP_6_ and phytohormones in governing seed vigor.

In addition to the role of IP_6_, the integration of phytohormone signals is also important for seed germination and seedling development [19]. For example, abscisic acid (ABA) controls storage reserve accumulation and desiccation tolerance of orthodox seeds, which lays the foundation for maintaining seed vigor before germination [20]. Auxin acts as a multi-functional regulator in many early developmental processes, including seed germination, subsequent root initiation, and elongation. Auxin also contributes to seed storability by stimulating abscisic acid (ABA) signaling [21]. Notably, the levels of indole-3-acetic acid (IAA) and the IAA/ABA ratio have been identified as core factors affecting germination and seedling development [22]. A recent study further revealed that OsGH3-2, an IAA-amido synthetase, influences seed storability and seed vigor by regulating ABA accumulation, which links auxin biosynthesis to the maintenance of seed vigor [23]. While these studies establish the critical role of auxin–ABA crosstalk in seed vigor, how IP_6_ metabolism connects to these hormone signaling pathways remains uncharacterized, especially at the epigenetic level.

Inositol polyphosphate kinase (IPK2) is a key component in the IP_6_ biosynthetic pathway. It catalyzes the conversion of Ins(1,4,5)P_3_ (IP_3_) to two IP_4_ isomers and Ins(1,3,4,5,6)P_5_ (IP_5_), both serving as precursors for IP_6_ biosynthesis [24]. The Arabidopsis genome encodes two IPK2 homologs, namely AtIPK2α and AtIPK2β [25]. Knockout of the *AtIPK2β* gene led to a 35% decrease in seed IP_6_ content in Arabidopsis, while the *atipk2α/atipk2β* double mutant was non-viable due to embryonic lethality [26]. Our earlier work demonstrated that *AtIPK2β* plays a critical role in alleviating glucose-mediated suppression of seed germination [27]. In rice, we previously reported that the inositol polyphosphate kinase gene (*OsIPK2*) exerts a negative regulatory role in gibberellin (GA)-mediated shoot elongation and fertility [28]. Furthermore, OsIPK2 functions as a chaperon of OsIAA11, a member of the AUX/IAA protein family, to suppress the auxin-dependent lateral root (LR) formation [29]. Notably, a single nucleotide mutation of *OsIPK2* resulted in reduced IP_6_ content at the seedling, tillering, and heading stages of rice [30]. Most recently, we revealed that *OsIPK2* plays a pivotal role in modulating phosphate (Pi) homeostasis and Pi-mediated root system architecture by reshaping inositol phosphate profiles [31]. However, its specific function in seed vigor and the underlying hormone crosstalk remains unclear.

In the present study, we demonstrated that exogenous IP_6_ treatment could markedly suppress seed vigor by blocking auxin signaling. RNA interference (RNAi) of *OsIPK2* in rice led to reduced seed IP_6_ content, elevated zinc (Zn) and iron (Fe) levels, and decreased germination rates. In contrast, the seedlings of *OsIPK2*-RNAi lines exhibited enhanced growth performance compared to the wild-type (WT). Furthermore, auxin-responsive genes were misregulated in the RNAi lines. Consistently, the H3K27me3 modification at the gene loci of these genes and the contents of endogenous hormones were altered in *OsIPK2*-silenced plants. Collectively, these findings provide novel insights into the molecular mechanisms by which *OsIPK2* coordinates nutrient storage and plant growth via hormone signaling, providing insights for improving seed vigor in rice genetic improvement programs.

## 2. Materials and Methods

### 2.1. Plant Materials and Growth Conditions

Seeds of Zhonghua11 (*Oryza sativa* L. ssp. *japonica*) were used for physiological experiments in this study. For the germination assay, 100 rice seeds were placed on Petri dishes saturated with different concentrations of IP_6_ (0, 10 μM, 100 μM, 1 mM, and 10 mM), followed by incubation at 30 °C and 80% relative humidity for 6 days. Each treatment group included three biological replicates. Germination was defined as the emergence of the radicle (length exceeding 2 mm) through the seed hull [32]. Germination rates were recorded daily starting from the 3rd day, and the data were statistically analyzed. The IP_6_ used in this study was 50% phytic acid aqueous solution (C_6_H_18_O_24_P_6_). Deionized water was used as the solvent for preparing IP_6_ solutions. The IP_6_ treatment solutions were renewed daily.

For the seedling phenotype assay, 30–50 rice seedlings were germinated at 30 °C and 80% relative humidity for 2 days, then cultivated in a growth chamber under a 14 h light/10 h dark cycle at 28 °C/24 °C for another 5 days. To investigate the effects of IP_6_ on seedling growth, germinated seedlings were treated with various concentrations of IP_6_ for 5 days. After treatments, the primary root length, shoot length, and fresh weight of each seedling were measured and recorded.

### 2.2. Vector Construction and Rice Transformation

To construct the pCAMBIA1302-*OsIPK2* vector, the coding sequence (CDS) of *OsIPK2* was amplified by PCR with the specific primers 5′-GCCATGGCCTCCGACCTGCGCCCG-3′ and 5′-CGAGATCTAGAATGATCTGAA GACG-3′ and inserted into the NcoI/BglII sites of the pCAMBIA1302 plasmid. The pCAMBIA1302-*OsIPK2* vector was transformed into rice callus by *Agrobacterium tumefaciens* (EHA105)-mediated transformation [33]. Homozygous T3 generation plants were used for subsequent experiments.

### 2.3. Histochemical Staining

*DR5::GUS* transgenic rice seeds at different germination stages were collected prior to histochemical staining. Samples were immersed in GUS staining solution (1 mg/mL X-Gluc, 100 mM phosphate buffer, 0.2% Triton X-100, and 10 mM EDTA) and incubated at 37 °C for 12 h in the dark. After staining, tissues were cleared through a gradient of 50–90% ethanol (*v*/*v*). GUS staining was observed using an Olympus SZX16 microscope (Olympus, Tokyo, Japan) with the bright-field optic.

For the TTC staining assay, seeds were immersed in a 0.1% 2,3,5-triphenyltetrazolium chloride (TTC) solution and incubated in the dark at 37 °C for 15 min. Subsequently, the seeds were washed with distilled water and observed under a light microscope.

### 2.4. Quantification of Phytate Content

The rice seeds were ground into fine powder and sieved. Phytic acid was extracted from 0.05 g samples using 0.6 M HCl as described previously [27]. The mixture was vortexed and shaken on a horizontal shaker for 2 h to facilitate extraction. After centrifugation at 8000× *g* and 25 °C for 10 min, 0.5 mL of the supernatant was collected. Subsequently, 0.5 mL sulfosalicylic acid–ferric chloride solution was added, and the mixture was incubated at 4 °C for 2 h. The absorbance of the reaction mixture was measured at 500 nm using a Synergy H1 Multi-Mode Microplate Reader (BIO-TEK Synergy H1, Winooski, VT, USA). Phytate concentration was calculated based on a standard curve generated with IP_6_ standard solution.

### 2.5. Quantification of Mineral Content

To characterize the mineral accumulation of seeds from wild-type (WT) and *OsIPK2*-RNAi transgenic lines (Ri-1, Ri-2), 0.3–0.5 g dry seeds from each genotype were treated with 5 mL nitric acid overnight, and digested at 160 °C for 4 h. After natural cooling, the solution was heated to evaporate the nitric acid to near dryness. The digest was washed three times with 1% nitric acid (*v*/*v*). A reagent blank was incubated in parallel. The elemental contents (Zn, Fe) of the test solution were determined using an Inductively Coupled Plasma-Mass Spectrometer (PE NexION 300, Waltham, MA, USA).

### 2.6. RNA Extraction and Quantitative Real-Time PCR

For transcript level analysis, total RNA was extracted from seedlings of wild-type and *OsIPK2*-RNAi transgenic rice using TRIZOL reagent (Invitrogen, Carlsbad, CA, USA) according to the manufacturer’s instruction. After being treated with DNase I (Fermentas, Burlington, ON, Canada) to remove genomic DNA, first-strand cDNA was synthesized using M-MLV Reverse Transcriptase reagent (Fermentas, Burlington, ON, Canada). qRT-PCR was performed on a StepOnePlus real-time PCR System (Applied Biosystems, Waltham, MA, USA) with SYBR Green Master Mix (TOYOBO, Osaka, Japan). *OsUBQ5* was used as an internal reference gene. The primers used here are listed in Table S1.

To clarify the spatiotemporal expression pattern of *OsIPK2*, we integrated and analyzed previously published transcriptomic and proteomic data from Li et al. [34]. This dataset provides comprehensive quantitative profiles of 14 major rice tissues. Transcriptomic data were normalized as transcripts per million (TPM) values, deposited in the National Center for Biotechnology Information (NCBI) Gene Expression Omnibus (GEO) with accession number GSE229334. Proteomic data were deposited in the ProteomeXchange Consortium via the PRIDE repository with dataset identifiers PXD041188 and PXD052005. In this study, we extracted the transcript and protein expression values of *OsIPK2* (LOC_Os02g32370) from the aforementioned dataset.

### 2.7. Chromatin Immunoprecipitation–Quantitative PCR Assays

Chromatin immunoprecipitation was performed as described previously [35]. The antibody used in ChIP assays was anti-trimethylated H3 (Lys 27) (Millipore (Burlington, MA, USA), 07-449). The promoter regions of *OsGH3.2, OsIAA9,* and *OsIAA20* were selected based on H3K27me3 ChIP-Seq profiles from the RiceENCODE database (V0.1, http://glab.hzau.edu.cn/RiceENCODE/index.html (accessed on 10 June 2024)) and genomic coordinates from the RGAP database (Release 7, https://rice.uga.edu/ index.shtml (accessed on 1 September 2025)). DNA fragments of *OsACTIN2* were used for normalization in ChIP-qPCR assays to detect enrichment of H3K27md3 modification. After reverse cross-linking and DNA purification, the enriched DNA was analyzed by qPCR with primers targeting the promoter regions of auxin-responsive genes. Fold enrichment was calculated relative to input DNA. The primers used for ChIP-qPCR and their sequences are listed in Table S1.

### 2.8. Bimolecular Fluorescence Complementation (BiFC) Assay

The full-length coding sequences of *OsIPK2* and *OsFVE* were amplified and inserted into the XbaI/KpnI sites of P35s-YFPn-Nos and P35s-YFPc-Nos vectors, respectively. Primers used in these experiments are listed in Table S1. Rice protoplast isolation and transfection were conducted as previously described [29]. Then, fluorescence in protoplasts was observed using a confocal laser scanning microscope (Olympus FV 1000, Tokyo, Japan).

### 2.9. Yeast Two-Hybrid Assay

Yeast two-hybrid (Y2H) assays were performed as previously described [29]. The full-length coding sequences of *OsIPK2* and *OsFVE* were inserted into the EcoRI/BamHI sites of pGADT7 and pGBKT7 vectors, respectively. The primer sequences used here are provided in Table S1. Bait and prey vector pairs were cotransformed into the yeast strain AH109 (Clontech, Mountain View, CA, USA) and incubated at 30 °C for 3 days on SD/-Leu/-Trp double-deficient (DDO) medium. Protein–protein interactions were detected using SD/-Leu/-Trp/-His/-Ade quadruple-deficient (QDO) medium.

### 2.10. Quantification of Phytohormones

The extraction and quantification of ABA and IAA were performed as previously described [28]. Briefly, 100 mg of 7-day-old rice seedling tissue was frozen in liquid nitrogen, ground into a fine powder, and spiked with 1.0 ng [^2^H_6_] ABA and [^2^H_5_] IAA as the internal standard for quantification, respectively. Ultra-high-performance liquid chromatography–tandem mass spectrometry (UHPLC-MS/MS) analysis was performed using an AB SCIEX 4500 triple quadrupole mass spectrometer coupled with a Shimadzu LC-30AD high-performance liquid chromatography (HPLC) system, which was equipped with dual 30AD pumps, SIL-30AC autosampler, a CTO-30A column oven, and a DGU-20A5R degasser. Each group included three biological replicates, with each replicate consisting of a pooled sample from 6 individual seedlings.

### 2.11. Microstructure Observation

To characterize the internal microstructure of mature rice seeds harvested from *OsIPK2*-RNAi transgenic lines (Ri-1, Ri-2) and WT plants, the cross-sections of seeds were observed using a Zeiss FE-SEM G300 scanning electron microscope (Zeiss, Jena, Germany) after sputter-coating with gold. For the observation of seed macroscopic morphology, images were acquired using a Zeiss Stemi 508 stereomicroscope (Zeiss, Jena, Germany). Three independent biological replicates were performed for each genotype.

### 2.12. Statistical Analysis

GraphPad Prism (Version 10.0) software and one-way analysis of variance (ANOVA) were employed to evaluate the overall significant differences in the measured indicators among different treatment groups, including germination rates, IP_6_-inhibited seedling growth parameters, mineral content, and phytate content. If the ANOVA result was significant (*p* < 0.05), Duncan’s multiple range test was further performed as a post hoc analysis to determine the specific pairwise differences between groups. The adjusted *p*-value from Duncan’s test was used to judge the significance of differences, with *p* < 0.05 considered statistically significant. For analyzing the fold change in gene expression levels, Student’s *t*-test was used to determine the significance of differences, with *p* < 0.05 considered statistically significant. All data are presented as the mean ± standard deviation (SD).

### 2.13. Accession Numbers

Genes analyzed in this study are available in GenBank with the following accession numbers: *O. sativa japonica OsIPK2* (Os02g32370), *OsGH3-2* (Os01g0764800), *OsIAA9* (Os02g0805100), *OsIAA20* (Os06g0166500), and *OsFVE* (Os01g0710000).

## 3. Results

### 3.1. Exogenous IP_6_ Inhibits Rice Seed Germination and Seedling Growth by Antagonizing Auxin Signaling

**Figure 1:**
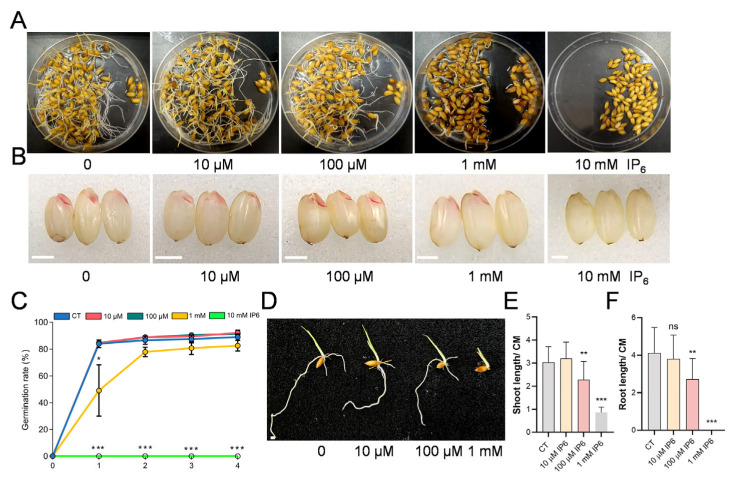
Exogenous IP_6_ inhibited seed germination and seedling growth. (**A**) Wild-type rice seeds germinated in plastic dishes following treatment with gradient IP_6_ concentrations (0, 10 μM, 100 μM, 1 mM, and 10 mM). (**B**) TTC staining for the viability of seeds treated with different concentration of IP_6_. Scale bars = 2 mm. (**C**) Germination rates (%) of rice seeds treated with different IP_6_ concentration over 4 days post-soaking. Data are mean ± SD (*n* = 3). Significant differences between each IP_6_-treated group and the control (0 μM) were determined by one-way ANOVA followed by Duncan’s multiple range test (* *p* < 0.05, *** *p* < 0.001). (**D**) Phenotype of 5 d old rice seedlings treated with 0, 10 μM, 100 μM, and 1 mM IP_6_. Scale bars = 2 mm. (**E**) Shoot length and (**F**) primary root length of WT seedlings under IP_6_ treatments. Data are mean ± SD (16 ≤ *n* ≤ 23). Asterisks indicate significant differences compared to the control by one-way ANOVA followed by Duncan’s multiple range test (** *p* < 0.01; *** *p* < 0.001; ns = not significant).

As a critical signaling molecule and major phosphorus reservoir in plant seeds, inositol hexakisphosphate (IP_6_) was known to regulate rice seedling vigor independently of environmental phosphorus [10]. To examine its dose-dependent effects on seed vigor, rice seeds were treated with varying concentrations of IP_6_ (0, 10 μM, 100 μM, 1 mM, and 10 mM) and germinated for 6 days. As shown in Figure 1A,C, high concentrations of IP_6_ (1 mM and 10 mM) severely inhibited seed germination, with the 10 mM treatment completely preventing germination. The inhibitory effect of IP_6_ on seed vigor was further verified by 2,3,5-triphenyl tetrazolium chloride (TTC) staining. Weak staining in seeds treated with high concentrations of IP_6_ indicates reduced embryonic metabolic activity (Figure 1B). At the seedling stage, IP_6_ application led to a significant suppression of growth in a concentration-dependent manner, including shortened primary root and impaired shoot development (Figure 1D–F). Collectively, these data demonstrate that exogenous IP_6_ disrupts early seedling establishment in rice in a concentration-dependent manner.

**Figure 2:**
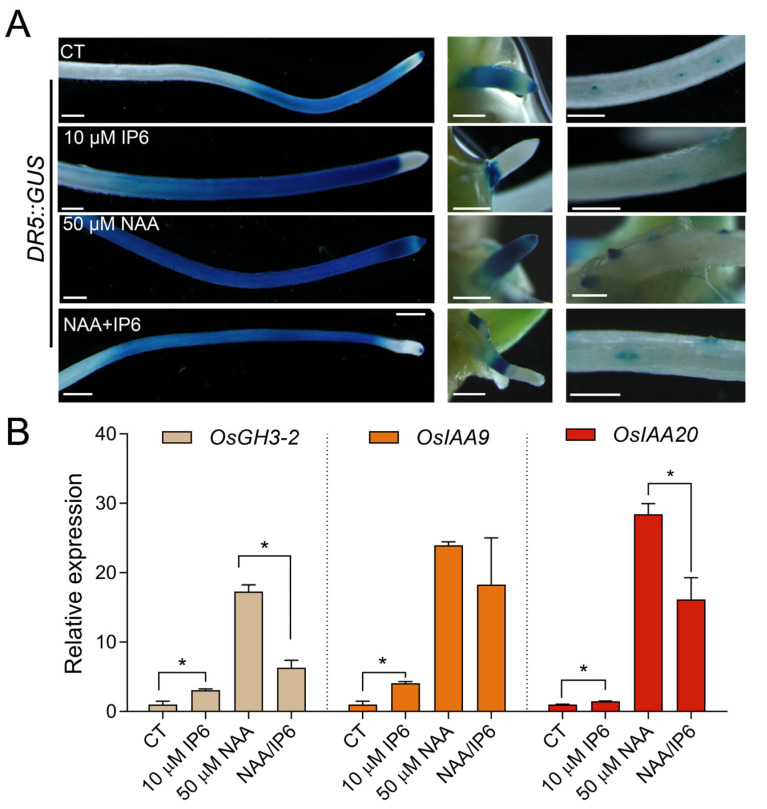
Exogenous IP_6_ treatment suppressed auxin response in rice seedlings. (**A**) *DR5::GUS* staining of rice seedlings under control (CT), 10 μM IP_6_, 50 μM NAA, or 10 μM IP_6_ and 50 μM NAA combined treatments, showing auxin signals in the primary root (**left**), coleoptile (**middle**), and lateral root (**right**). Scale bars = 1 mm. (**B**) Relative expression levels of *OsGH3-2*, *OsIAA9*, and *OsIAA20* genes under the above treatments. Data are mean ± SD (*n* = 3). Asterisks indicate significant differences compared to the control by Student’s *t*-test (* *p* < 0.05).

Auxin is a key plant hormone that regulates seed dormancy and germination. To clarify how IP_6_ modulates plant growth, we investigated the effect of IP_6_ on auxin signaling using a *DR5::GUS* reporter system (Figure 2). GUS staining revealed that auxin signal was mainly distributed in the primary root (PR), crown root tips, and lateral root primordia (Figure 2A), a pattern consistent with previous observations [36]. Notably, 10 μM IP_6_ treatment suppressed the signal in crown root tips and lateral root primordia. In contrast, 50 μM synthetic auxin NAA treatment promoted the accumulation of auxin signal, particularly in emerging lateral roots. Furthermore, when co-treated with IP_6_ and NAA, auxin signals in the tips of PR, crown root (CR) and lateral root (LR) were markedly suppressed. It suggests that IP_6_ appeared to alleviate NAA-induced *DR5:GUS* expression in root apices. The observation that IP_6_-mediated suppression of *DR5:GUS* expression shared phenotypic similarity with the effects of the auxin transport inhibitor NPA or mutations in auxin influx carrier genes, suggesting that IP_6_ might disrupt auxin transport or signaling at the tissue spatial level [37,38].

This spatial observation of IP_6_-mediated inhibition of auxin signaling was further validated by quantitative RT-PCR analysis. *OsGH3-2*, *OsIAA9*, and *OsIAA20* are early auxin-responsive genes, which are strongly induced by auxin treatment [23,39]. To detect the effects of NAA and IP_6_ on the transcription of these genes, we performed qRT-PCR analysis. Consistent with the *DR5::GUS* staining results, qRT-PCR results showed that IP_6_ alone could induce the expression of these auxin early-responsive genes, while NAA markedly activated the transcription (Figure 2B). However, when seedlings were co-treated with NAA and IP_6_, the induction effects of auxin were attenuated, implying that IP_6_ can antagonize auxin-induced gene expression. Collectively, these data suggest that IP_6_ functions antagonistically to auxin by suppressing auxin’s apical distribution or biosynthesis, thereby inhibiting seed germination and seedling emergence.

### 3.2. Spatiotemporal Expression Pattern of OsIPK2 Suggests Its Role in Early Development

**Figure 3:**
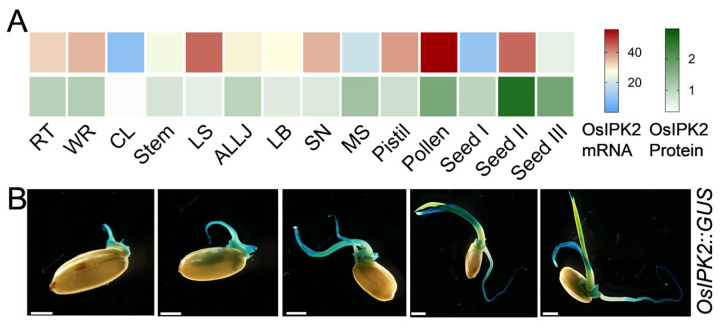
Tissue-specific expression profile of *OsIPK2* during seed germination. (**A**) Expression pattern of *OsIPK2* in diverse rice tissues, including root tip (RT), whole root (WR), culm and leaves (CL), stem, leaf sheath (LS), auricle, ligule and lamina joint (ALLJ), leaf blade (LB), spike neck (SN), mature spikelet (MS), pistil, pollen, early stage immature seeds (seed I), grain filling seeds (seed II), and mature seeds (seed III). The heatmaps were generated using transcriptomic and proteomic data from Li et al. [34]. (**B**) GUS staining driven by the *OsIPK2* promoter during seed germination and early seedling development stages. Scale bars = 2 mm.

Rice inositol polyphosphate kinase (OsIPK2) is a key enzyme in the IP_6_ biosynthetic pathway [30]. To clarify the spatiotemporal expression pattern of *OsIPK2* during seed germination, we analyzed its tissue-specific expression profiles using previously published multi-omics data, which systematically quantified the mRNA and protein levels of over 15,000 rice genes across 14 major tissues via RNA-seq and tandem mass tag (TMT)-based quantitative proteomics [34]. The heatmap revealed that *OsIPK2* is widely expressed in various rice tissues (Figure 3A). Notably, both *OsIPK2* mRNA and protein levels are particularly elevated in developing seeds and young seedlings. Histochemical staining of *OsIPK2::GUS* transgenic seedlings further confirmed this expression pattern (Figure 3B). GUS staining intensified throughout seed germination and early seedling development, and prominent expression was observed in the primary root and coleoptile as germination progressed. Collectively, these data indicate that *OsIPK2* plays a critical role in regulating seed vigor and early seedling development.

### 3.3. OsIPK2 Is a Key Regulator of Seed Morphology, IP_6_ Biosynthesis, and Mineral Contents

**Figure 4:**
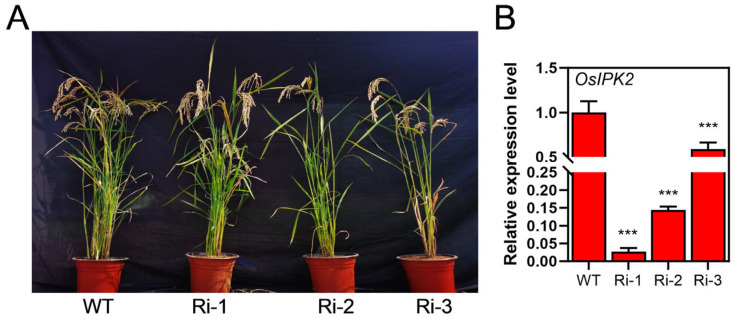
Identification of RNA interference (RNAi) lines of the *OsIPK2* gene in rice. (**A**) The phenotypes of wild-type (WT) and *OsIPK2*-RNAi transgenic lines (Ri-1, Ri-2, Ri-3). (**B**) Relative expression levels of *OsIPK2* determined by qRT-PCR. *OsUBQ5* was used as an internal control. Data shown are the mean ± SD of three replicates. Asterisks represent extremely significant differences compared to the WT by Student’s *t*-test (*** *p* < 0.001).

Homozygous mutants of the *IPK2* gene in Arabidopsis and rice are unavailable for functional studies due to severe fertility defects, which limits their use in genetic analyses [26,31]. To investigate the function of *OsIPK2*, we generated transgenic rice plants overexpressing *OsIPK2* driven by the CaMV 35S promoter (Figure 4A). Unexpectedly, qRT-PCR analysis of three independent transgenic lines showed a significant reduction in *OsIPK2* mRNA accumulation, indicating post-transcriptional gene silencing (PTGS) (Figure 4B). Consequently, these independent lines effectively served as *OsIPK2*-RNA interference (RNAi) lines and were renamed as Ri-1, Ri-2, and Ri-3. Among them, lines Ri-1 and Ri-2, which showed the strongest silencing effect, were selected for subsequent phenotypic and molecular analyses.

**Figure 5:**
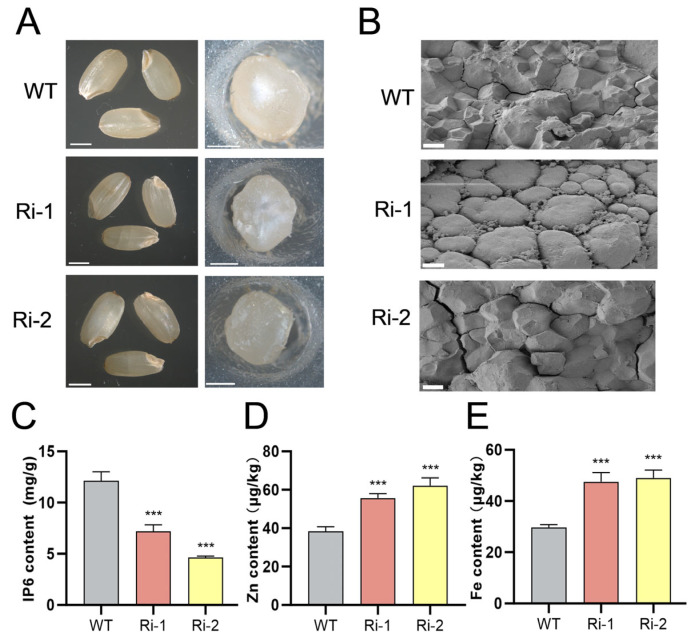
Phenotypic and biochemical characterization of WT and *OsIPK2*-RNAi transgenic rice seeds. (**A**) Seed morphology of WT, Ri-1, and Ri-2 lines (**left panel**, whole seeds; **right panel**, cross-sections of seeds). Scale bars = 1 mm. (**B**) Scanning electron microscopy (SEM) images of the cross-sections of seeds. Scale bars = 5 μm. (**C**–**E**) IP_6_ (**C**), Zn (**D**), and Fe (**E**) contents in WT, Ri-1, and Ri-2. Data are presented as mean ± SD (*n* = 3). *** *p* < 0.001 indicates extremely significant differences compared to WT (Duncan’s multiple range test).

Given the relevance of seed traits to vigor, we examined the seed morphology of WT and *OsIPK2*-RNAi lines (Ri-1, Ri-2) via bright-field microscopy. WT grains were translucent, plump, and smooth, with a regularly rounded transverse cross-section. In contrast, RNAi lines showed reduced translucency and slight shrinkage (Figure 5A). We further analyzed the microstructural characteristics of the seeds using scanning electron microscopy (SEM). The cross-section of WT seeds exhibited polygonal endosperm cells with relatively distinct cell boundaries and a surface without obvious cracks (Figure 5B). In contrast, the endosperm of the Ri-1 line showed a smoother surface with rounded, closely packed cells. For Ri-2, its endosperm surface was rough and cracked, with significantly distorted cellular structures. Collectively, these structural abnormalities indicate that *OsIPK2* is required for normal rice grain development, which may directly influence seed vigor.

To analyze the role of *OsIPK2* in seed IP_6_ accumulation, we measured the levels of IP_6_ and minerals in seeds of WT and *OsIPK2*-RNAi lines. The results showed that RNAi lines exhibited lower IP_6_ content compared to WT seeds (Figure 5C). In contrast, they accumulated higher levels of iron (Fe) and zinc (Zn) compared to WT (Figure 5D,E). This inverse correlation between seed IP_6_ content and mineral accumulation is consistent with previous studies on low-phytic-acid mutants, which demonstrate that reducing IP_6_ levels can enhance the accumulation of essential minerals in rice grains [40]. Therefore, our results suggest that impaired IP_6_ biosynthesis mediated by *OsIPK2* knockdown disrupts mineral storage homeostasis, and further confirm the essential catalytic function of OsIPK2 in vivo.

### 3.4. Knockdown of OsIPK2 Results in Reduced Seed Germination Rate and Promoted Seedling Development

**Figure 6:**
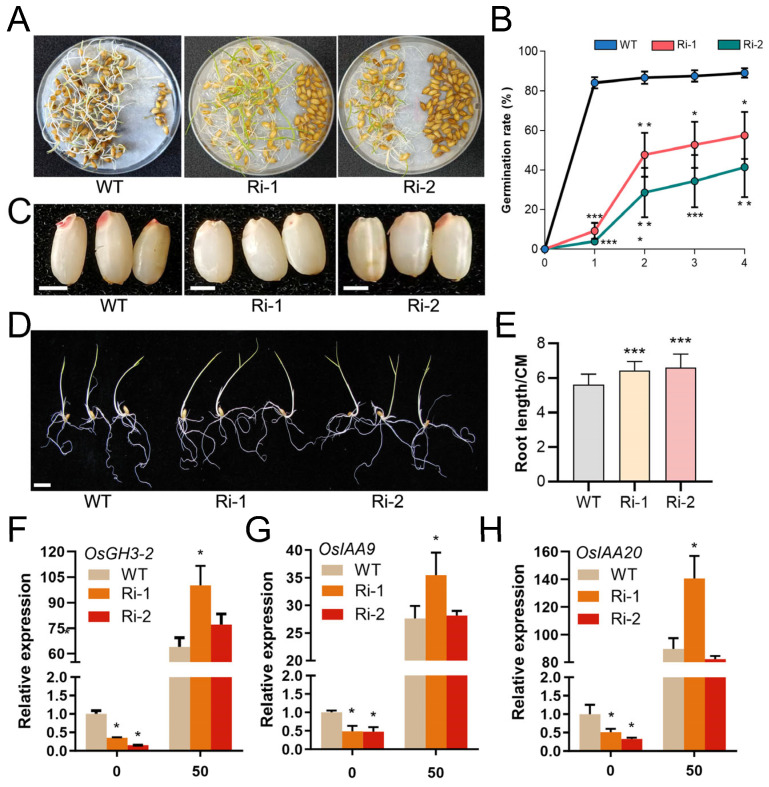
Germination and seedling growth characteristics of wild-type (WT) and *OsIPK2*-RNAi transgenic rice seeds. (**A**) The 4 d old germinated seeds of *OsIPK2*-RNAi and WT following 48 h of soaking. (**B**) Germination rates (%) of *OsIPK2*-RNAi lines and WT over 4 days post-soaking. Significant differences were observed between transgenic lines and WT (Student’s *t*-test: * *p* < 0.05, ** *p* < 0.01, *** *p* < 0.001). Data are mean ± SD (*n* = 3). (**C**) TTC staining to assess seed viability of RNAi lines and WT. Scale bars = 2 mm. (**D**) Phenotype of 5 d old *OsIPK2*-RNAi and WT rice seedlings. Scale bars = 1 cm. (**E**) Primary root lengths of *OsIPK2*-RNAi and WT rice seedlings. Data are presented as mean ± SD (*n* ≥ 18). *** *p* < 0.001 indicates extremely significant compared to WT (Duncan’s multiple range test). (**F**–**H**) Relative expression levels of *OsGH3-2* (**F**), *OsIAA9* (**G**), and *OsIAA20* (**H**) genes in WT, Ri-1, and Ri-2 under 50 μM NAA treatment. Data are mean ± SD (*n* = 3). * *p* < 0.05 indicates significant difference compared to WT (Student’s *t*-test).

To evaluate the impact of impaired IP_6_ accumulation on seed vigor, we examined the germination kinetics of *OsIPK2*-RNAi lines and WT seeds over a 4-day period following 48 h of soaking. As illustrated in Figure 6A,B, the final germination rate of WT seeds reached approximately 85% by day 4. In contrast, the two *OsIPK2*-RNAi lines exhibited significantly delayed and reduced germination. Their germination rates were notably lower than that of WT at each time point, and germination was significantly delayed. 2,3,5-triphenyltetrazolium chloride (TTC) staining assays further confirmed these observations (Figure 6C). The seeds of *OsIPK2*-RNAi lines showed weaker TTC staining, indicating compromised seed viability. Collectively, these results demonstrate that knockdown of *OsIPK2* markedly impairs seed viability and delays germination.

Furthermore, *OsIPK2*-RNAi lines displayed enhanced root elongation under IP_6_ treatment (Figure 6D,E). Notably, the root-growth-promoting phenotype was consistent with the growth inhibition phenotype observed in *OsIPK2*-overexpressing lines [29]. This suggests that *OsIPK2* acts as a negative regulator in seedling development, but is necessary for seed germination.

To investigate whether *OsIPK2* is involved in the regulation of auxin-responsive genes, we analyzed the expression of *OsGH3-2*, *OsIAA9,* and *OsIAA20* in *OsIPK2*-RNAi lines (Figure 6F–H). In the absence of exogenous auxin treatment, the transcript levels of these genes in RNAi lines were significantly lower than those in the WT, suggesting that basal auxin signaling may be attenuated or endogenous auxin levels reduced in the RNAi background. Under NAA treatment, these genes were induced in all genotypes, but their expression levels were significantly higher in Ri-1 and Ri-2 than in WT. This hyper-induction of auxin-responsive genes suggests that silencing *OsIPK2* enhances sensitivity to auxin, potentially by impairing auxin-dependent feedback regulatory mechanisms. *OsGH3-2*, *OsIAA9*, and *OsIAA20* have been reported to function in root architecture, germination, and environmental stress adaptation [41,42,43,44]. For example, *OsGH3-2*, which conjugates free IAA into inactive amino acid conjugates, may be upregulated to compensate for elevated free IAA levels. In contrast, *OsIAA9* and *OsIAA20*, as Aux/IAA repressors, may show increased expression to attenuate auxin responses, consistent with their roles in modulating plant growth and stress adaptation.

### 3.5. OsIPK2 Influences H3K27me3 Repressive Marks on Auxin-Responsive Gene Loci

**Figure 7:**
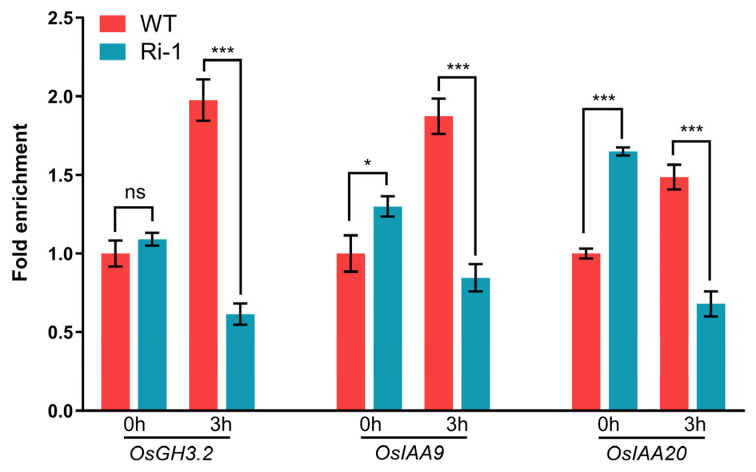
RNA interference of *OsIPK2* affected H3K27me3 enrichment at the gene loci of *OsGH3-2*, *OsIAA9*, and *OsIAA20* in response to auxin. ChIP-qPCR analysis of fold enrichment of H3K27me3 at the promoter regions (selected via RiceENCODE and RGAP databases) of *OsGH3-2*, *OsIAA9*, and *OsIAA20* in WT and Ri-1 with or without 50 μM NAA treatment for 3 h. Data are presented as mean ± SD (*n* = 3). Asterisks indicate significant differences compared to WT (Student’s *t*-test: * *p* < 0.05, *** *p* < 0.001; ns = not significant).

Since chromatin modification is an important mechanism for fine-tuning transcriptional responses during seed germination and LR formation, we wondered if *OsIPK2* affects the repressive histone mark H3K27me3 at the loci of these auxin-responsive genes [45,46]. Thus, chromatin immunoprecipitation (ChIP)-qPCR analyses were performed to identify the H3K27me3 enrichment at the promoter regions of three auxin-responsive genes. A single region for each gene locus was selected for subsequent detailed analysis. Under basal conditions, Ri-1 exhibited higher H3K27me3 enrichment at the *OsIAA9* and *OsIAA20* gene loci compared to WT. Under NAA treatment for 3 h, H3K27me3 levels at each gene locus were significantly reduced in Ri-1 compared with that of WT (Figure 7). In contrast, WT plants displayed relatively stable H3K27me3 marking in response to auxin induction. Taken together, our data demonstrate that knockdown of *OsIPK2* leads to hyperactivation of auxin-responsive genes. This effect is likely mediated, at least in part, by a decrease in the repressive H3K27me3 mark at their chromatin loci, suggesting that *OsIPK2* functions to attenuate the auxin response by promoting repressive histone methylation-based gene silencing.

Our previous study in Arabidopsis demonstrated that AtIPK2β interacts with FVE and HDA6 to reduce H3K27me3 levels at the FLC locus [35]. As a rice homolog of Arabidopsis FVE, overexpression of *OsFVE* can partially complement the flowering phenotype of the *fve* loss-of-function mutant in Arabidopsis [47]. Thus, we hypothesized that OsIPK2 might similarly interact with OsFVE. To test this, we performed bimolecular fluorescence complementation (BiFC) and yeast two-hybrid (Y2H) assays (Figure S1). Surprisingly, no direct interaction was detected between OsIPK2 and OsFVE. These results suggest that while the role of *IPK2* in modulating H3K27me3 is functionally conserved, the specific protein–protein interaction mechanism involving FVE has diverged between Arabidopsis and rice.

### 3.6. OsIPK2 Plays a Role in Auxin Homeostasis of Rice Seedlings

**Figure 8:**
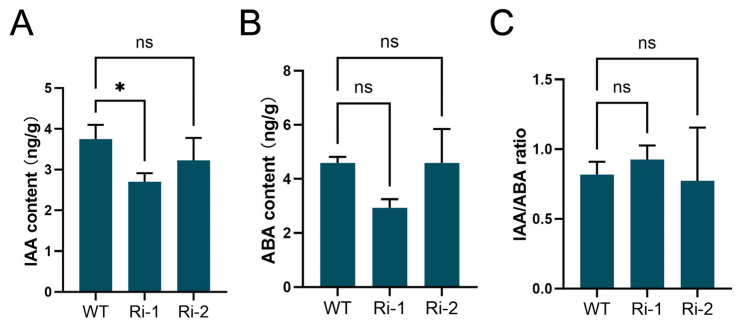
Endogenous auxin and ABA contents in WT and *OsIPK2*-RNAi transgenic rice seedlings. (**A**) Endogenous auxin contents in WT, Ri-1, and Ri-2 seedlings. (**B**) Endogenous ABA contents in WT, Ri-1, and Ri-2 seedlings. (**C**) The ratio of IAA to ABA in WT, Ri-1, and Ri-2 seedlings. Data are presented as mean ± SD (*n* = 3). Asterisks indicate significant difference compared to WT by one-way ANOVA followed by Duncan’s multiple range test (* *p* < 0.05). ns, not significant.

*OsGH3-2* is known to function in maintaining IAA homeostasis and regulating seed germination processes [41]. To assess the potential impact of *OsIPK2* on phytohormone balance, we quantified the endogenous levels of IAA and ABA in *OsIPK2*-RNAi lines. As shown in Figure 8A, the IAA content in Ri-1 was significantly lower than that in WT plants, whereas no significant difference was detected in Ri-2. A similar trend was detected for ABA accumulation; however, no significant differences were observed between each RNAi line and WT (Figure 8B). In addition, the endogenous IAA/ABA ratio remained unchanged between RNAi lines and WT (Figure 8C). These findings suggest that *OsIPK2* plays a critical role in auxin homeostasis during seed germination and seedling development.

## 4. Discussion

Inositol hexaphosphate (IP_6_, phytic acid) is the most abundant inositol phosphate compound in plants. It serves as a major phosphorus reservoir in seeds and also acts as a natural antioxidant. In the food industry, IP_6_ is used to reduce the browning of fruit and vegetables by inhibiting the activities of polyphenol oxidase (PPO) and peroxidase (POD) [48]. Interestingly, exogenous application of IP_6_ has long been observed to inhibit seed germination, suggesting its potential to prolong dormancy and enhance seed storability. In this study, we confirmed that high concentrations of IP_6_ exert a phytotoxic effect on seed germination and seedling growth in rice (Figure 1A). Furthermore, our data revealed that IP_6_ treatment disrupted NAA-induced *DR5::GUS* expression in the transgenic rice and suppressed the expression of auxin-responsive genes (Figure 2). Previous studies have showed that elevated auxin biosynthesis promoted ABA responses and suppressed seed germination, while mutations in auxin biosynthetic enzyme YUCCA (YUC) or TIR1/AFB receptors strongly inhibited ABA signaling and seed dormancy [21]. Given that auxin is a positive regulator of ABA-mediated dormancy, the disruption of auxin signaling by IP_6_ would theoretically attenuate ABA signaling and thereby promote germination. However, we observed the opposite outcome. This paradox indicates that IP_6_ does not simply inhibit auxin signaling. The underlying molecular mechanism remains largely unclear. Since IP_6_ cannot be directly absorbed by plants and requires hydrolysis via phytase, its inhibitory effect likely involves signal perception rather than nutritional utilization. Auxin is well recognized for promoting coleoptile elongation and rapid seedling establishment throughout germination and early post-germinative development [49]. Thus, IP_6_-mediated disruption of auxin signaling could directly impair these critical early developmental processes, resolving the earlier paradox by providing a plausible mechanism for the germination-inhibitory effect of IP_6_. In addition, future measurements of IP_6_ uptake efficiency, tissue-specific IP_6_ concentrations, and phytase activity in IP_6_-treated seeds and seedlings will help elucidate this underlying mechanism.

Contrary to the inhibitory effects of exogenous IP_6_ on seed germination, accumulating evidence from wheat, rice, and legumes indicates that seeds with high phytic acid content germinate earlier and grow faster for more vigorous seedlings [50]. For instance, homozygous nonsense mutants of *OsIPK1*, a key rate-limiting enzyme for IP_6_ synthesis, fail to germinate entirely [12]. In the inositol phosphate metabolic pathway, IPK2 provides the substrate IP_5_ for IPK1 to produce IP_6_ [25]. Our data showed that *OsIPK2-RNAi* lines exhibited a significant reduction in seed IP_6_ content and an increased mineral bioavailability, suggesting that seed-derived IP_6_ synthesized via OsIPK2 is essential for efficient seed germination (Figure 5C–E and Figure 6A–C). This aligns with our previous findings in Arabidopsis, where the *atipk2β* mutant is hypersensitive to environmental signals that inhibit germination, which could be rescued by exogenous application of IP_6_ synthesis precursors [27]. These results support a positive correlation between endogenous seed phytate content and germination performance, indicating that *OsIPK2* is a key regulator of seed germination and early seedling establishment through its conserved function in IP_6_ biosynthesis. Furthermore, a recent study revealed that IPK2-type kinases possess 4/6-InsP7 synthase activity, which is critical for enhancing the DNA-binding capacity of Heat Shock Factors (HSFs) during stress responses [25]. This additional catalytic activity of IPK2 highlights its roles in plant physiology and identifies it as a potential molecular target for improving seed vigor and stress resilience in crop breeding.

Regarding seedling development, *OsIPK2*-RNAi seedlings exhibited enhanced growth and altered expression patterns of auxin-responsive genes (Figure 6D,E), a phenotype opposite to the growth inhibition caused by exogenous IP_6_ (Figure 1D–F). Our previous work demonstrated that OsIPK2 functions as a co-repressor of Aux/IAA proteins in the auxin signaling pathway, suppressing lateral root formation and acting as a negative regulator of Pi homeostasis [29,31]. Based on these findings, we propose that silencing *OsIPK2* may enhance seedling growth by modulating the activity of Aux/IAA repressors and altering phosphate homeostasis, thereby integrating the effects of IP_6_ deficiency to promote seedling development.

As a repressive histone mark, Histone H3 lysine 27 trimethylation (H3K27me3) plays an important role in regulating auxin signaling [51]. ChIP-chip assays of H3K27me3 in Arabidopsis have showed that H3K27me3 directly targets gene families across the entire auxin pathways, including 14 Arabidopsis *AUX/IAA* genes and 8 PIN auxin efflux carriers, leading to their transcriptional repression. In the present study, we revealed that *OsIPK2* influenced H3K27me3 deposition at the loci of auxin-responsive genes in rice, particularly under NAA treatment (Figure 7). These dynamic changes in the epigenetic landscape likely contributed to the hyperactivation of these genes in response to auxin (Figure 6F–H). The molecular mechanism by which *OsIPK2* influences H3K27me3 deposition remains unclear. To investigate whether the regulatory mechanism of IPK2 in histone modification is conserved between Arabidopsis and rice [35], we tested the potential interaction between OsIPK2 and OsFVE, a key component of epigenetic repressive complexes (Figure S1). In contrast to the previously observed AtIPK2β-AtFVE interaction in Arabidopsis, no interaction was detected between OsIPK2 and OsFVE in rice. Given that inositol phosphates can directly influence chromatin-modifying complexes [52], we propose that *OsIPK2* may affect H3K27me3 deposition through an FVE/HDA6-independent pathway, potentially involving the production of specific inositol phosphate species. Future studies should aim to identify the IP molecules involved and elucidate how they interact with the epigenetic machinery to fine-tune H3K27me3 levels in response to auxin signals.

Based on these findings, we propose a model in which *OsIPK2* regulates seed vigor-related traits by acting as a central integrator of inositol phosphate metabolism, auxin signaling, and epigenetic regulation. The reduced free IAA content in the Ri-1 line (Figure 8A), together with the hypersensitive induction of auxin-responsive genes upon NAA treatment (Figure 6F–H), reflects a disruption of auxin signaling homeostasis. This altered sensitivity is associated with *OsIPK2*-dependent dynamics of H3K27me3 deposition on the chromatin of these genes (Figure 7). Furthermore, we observed that *OsIPK2* modulates the expression of *OsGH3-2* (Figure 6F), which encodes an IAA-amido synthetase and is well-documented as a negative regulator of seed storability [23]. Our data suggest that *OsIPK2* modulates auxin homeostasis, at least partially, by regulating *OsGH3-2*, thereby influencing key seed physiological traits (Figure 8). This positions *OsIPK2* not only as an enzyme in IP_6_ synthesis, but as a critical regulator at the core of the interconnected metabolic and signaling networks that determine seed performance.

The regulatory model of *OsIPK2* highlights its potential as a molecular target for rice improvement. Specifically, the elevated Zn/Fe accumulation in *OsIPK2*-RNAi lines provides a promising direction for biofortification. However, *OsIPK2*-RNAi lines also exhibit reduced germination rate and abnormal endosperm structure. These drawbacks are tightly linked to IP_6_ deficiency. For practical breeding, seed-specific silencing of *OsIPK2* or seed priming with a low-concentration inorganic phosphate (Pi) solution may mitigate these adverse traits.

## 5. Conclusions and Limitations

In conclusion, our findings demonstrate that *OsIPK2* is an important regulator of seed vigor-related traits in rice, including germination and early seedling establishment. Our results indicate that exogenous application of IP_6_ inhibits seed germination and seedling growth, at least in part by suppressing the auxin response. Conversely, RNAi-mediated knockdown of *OsIPK2* led to reduced seed IP_6_ content, enhanced mineral bioavailability, and altered auxin content. Notably, we found that *OsIPK2* influences H3K27me3 deposition on auxin-responsive genes, providing a potential mechanism for regulating auxin sensitivity and seed vigor. Future studies should aim to identify the specific inositol phosphate molecules involved and elucidate their direct molecular targets. Understanding these regulatory mechanisms may facilitate the development of strategies to improve seed quality in rice and other cereal crops.

In addition, this study has several inherent limitations. First, we only used a single japonica rice genotype (Zhonghua11) for all experiments. Genetic background can significantly influence traits related to phytic acid metabolism, hormone homeostasis, and seed vigor, so the regulatory role of *OsIPK2* observed here may not be fully generalizable to indica or other rice subspecies. Second, our functional analysis relied exclusively on RNAi-mediated silencing lines. The incomplete silencing could influence phenotypic interpretation. Third, we focused on IAA and ABA levels but did not quantify other phytohormones that may interact with auxin/ABA pathways during seed germination. Fourth, for epigenetic analysis, we only examined H3K27me3 modification of selected auxin-responsive genes, without exploring other histone modifications or DNA methylation that could contribute to OsIPK2-mediated regulation. Direct in vivo interaction or binding evidence between OsIPK2 and epigenetic regulators remains unknown. Fifth, our seed phenotype analysis was limited to controlled growth conditions without conducting accelerated aging tests, temperature treatments, or stress conditions, and the agronomic performance of *OsIPK2*-RNAi lines under natural field conditions remains unevaluated. In addition, the ionic composition or slight osmotic pressure changes at different IP_6_ concentrations were not explicitly evaluated.

## Data Availability

The original contributions presented in this study are included in the article/Supplementary Materials. Further inquiries can be directed to the corresponding authors.

## Supplementary Materials

The following supporting information can be downloaded at: https://www.mdpi.com/article/10.3390/biology15020155/s1, Figure S1: Protein–protein interaction assays of OsIPK2-OsFVE. (A) The bimolecular fluorescence complementation (BiFC) assay to detect the OsIPK2–OsFVE interaction. nYFP-OsIPK2 and cYFP-OsFVE were co-expressed in rice protoplasts. Scale bar = 5 μm. (B) Yeast two-hybrid assays to detect the OsIPK2 -OsFVE interaction. OsFVE was fused to the GAL4 activation domain (AD) as prey and OsIPK2 was fused to the GAL4 DNA-binding domain (BD) as bait. The interactions were examined on the double dropout (DDO) medium (SD/-Leu/-Trp) and quaternary dropout (QDO) medium (SD/-Ade/-His/-Leu/-Trp); Table S1: Sequences of primers used in this work.

## Abbreviations

The following abbreviations are used in this manuscript:

IP_6_	Inositol Hexakisphosphate
IP_3_	Ins(1,4,5)P_3_
IP_5_	Ins(1,3,4,5,6)P_5_
IPK2	Inositol Polyphosphate Kinase
GUS	β-Glucuronidase
IAA	Indole-3-Acetic Acid
ABA	Abscisic Acid
NAA	1-Naphthaleneacetic Acid
TTC	2,3,5-Triphenyltetrazolium Chloride
qRT-PCR	Quantitative Real-Time Polymerase Chain Reaction
ChIP	Chromatin Immunoprecipitation
SEM	Scanning Electron Microscopy
