# Supplementary material for: OsIPK2 Regulates Seed Vigor by Integrating IP6 Biosynthesis, Auxin Signaling, and H3K27me3 Deposition in Japonica Rice
Source: Biology (Basel). 2026 Jan 15;15(2):155. doi: 10.3390/biology15020155 (PMC12837329; doi:10.3390/biology15020155)
Supplement: Supplementary file 1 [file biology-15-00155-s001.zip › Figure S1.pdf]

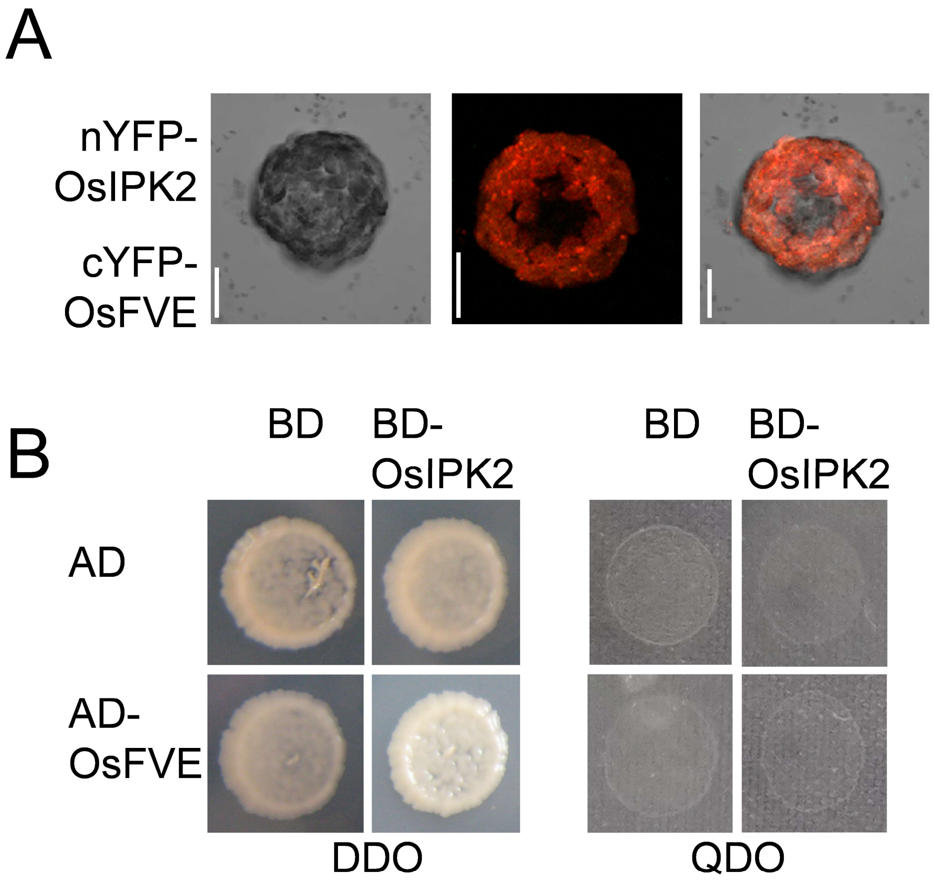


**Figure S1.** Protein-protein interaction assays of OsIPK2-OsFVE. (A)The bimolecular fluorescence complementation (BiFC) assay to detect the OsIPK2 -OsFVE interaction. nYFP-OsIPK2 and cYFP-OsFVE were co-expressed in rice protoplasts. Scale bar = 5 μm. (B) Yeast two-hybrid assays to detect the OsIPK2 -OsFVE interaction. OsFVE was fused to GAL4 activation domain (AD) as prey and OsIPK2 was fused to GAL4 DNA-binding domain (BD) as bait. The interactions were examined on the double dropout (DDO) medium (SD/-Leu/-Trp) and quaternary dropout (QDO) medium (SD/-Ade/-His/-Leu/-Trp).
