# Supplementary material for: OsIPK2 Regulates Seed Vigor by Integrating IP6 Biosynthesis, Auxin Signaling, and H3K27me3 Deposition in Japonica Rice
Source: Biology (Basel). 2026 Jan 15;15(2):155. doi: 10.3390/biology15020155 (PMC12837329; doi:10.3390/biology15020155)
Supplement: Supplementary file 1 [file biology-15-00155-s001.zip › Table S1.pdf]

Table S1. Sequences of primers used in this work.

Name	Primer Sequence (5' to 3')
Primers for quantitative real-time PCR	
OsIPK2 RT-F	AGGAGCAAACCCTGTACCACTTCT
OsIPK2 RT-R	AAGTCCACCAGCTTCACCCTTACA
UBQ5 RT-F	ACCACTTCGACCGCCACTACT
UBQ5 RT-R	ACGCCTAAGCCTGCTGGTT
OsGH3-2 RT-F	CGAGGAGGTGATGGACTACG
OsGH3-2 RT-R	GGCTGAAGTGCTTGGAGATG
OsIAA9 RT-F	CAACATATGGAGCTGGAGCTTGGGCT
OsIAA9 RT-R	CAACTTAAGTTAACCCAGTATCTTCAGGC
OsIAA20 RT-F	TGGGCAACAAGAGGAGGAAG
OsIAA20 RT-R	CGTATGAGCCGAGGATGGA
Primers for ChIP-qPCR	
OsGH3-2 ChIP-F	TTTCAGTCCCTGTCCACTTACCATT
OsGH3-2 ChIP-R	TGTTTTGTTGCTGCTTGGGTATGTG
OsIAA9 ChIP-F	TGAAGGTGAAGAAGGAAGGCGAC
OsIAA9 ChIP-R	AGCTACTGACCTTGGTGGTCGTTG
OsIAA20 ChIP-F	ATCGTCTTGCATTGCTCTCGATC
OsIAA20 ChIP-R	AGCTCCATCCGTCTCTCCAATTCA
OsACT2 ChIP-F	TCTTACGGAGGCTCCACTTAAC
OsACT2 ChIP-R	TCCACTAGCATAGAGGGAAAGC
Primers for Y2H	
OsIPK2-EcoRI-F	AAGAATTCATGGCCTCCGACCTGCGC
OsIPK2-BamHI-R	AAGGATCCAGAATGATCTGAAGA
OsFVE-EcoRI-F	AAGAATTCATGAAGGAGAAGGGGTCG
OsFVE-BamHI-R	AAGGATCCTCAGCTCCTTGGAGCACA
Primers for BiFC	
OsIPK2-XbaI-F	AATCTAGAATGGCCTCCGACCTGCGC
OsIPK2-KpnI-R	AAGGTACCTCAAGAATGATCTGAAGA
OsFVE-XbaI-F	AATCTAGAATGAAGGAGAAGGGGTCG
OsFVE-KpnI-R	AAGGTACCTCAGCTCCTTGGAGCACA
